# Should We Worry About Spillover Effects of Sugar Sweetened Beverage Taxation Policies? Comment on "Understanding Marketing Responses to a Tax on Sugary Drinks: A Qualitative Interview Study in the United Kingdom, 2019"

**DOI:** 10.34172/ijhpm.2023.7793

**Published:** 2023-06-06

**Authors:** Peter Hangoma, Mwimba Chewe

**Affiliations:** ^1^Department of Health Policy and Management, School of Public Health, University of Zambia, Lusaka, Zambia; ^2^Chr. Michelsens Institute (CMI), Bergen, Norway; ^3^Bergen Centre for Ethics and Priority Setting in Health (BCEPS), University of Bergen, Bergen, Norway

**Keywords:** Sugar-Sweetened Beverage Tax, Non-communicable Diseases, Economics of Taxation

## Abstract

Taxes on sugar sweetened beverages (SSBs) have been widely implemented and heralded as a panacea in reversing the growing burden of non-communicable diseases (NCDs). Using a qualitative research methodology, Forde et al explored how sugary drink companies respond to changes in taxation positing that relative effectiveness of sugar taxes will not only depend on how prices are affected, and how consumers respond, but also how producers respond by reformulating their products or engaging in counteractive marketing strategies. They argue that these responses may undermine the public health goal. We discuss some of the key issues that arise in their paper and conclude that company responses may not be sufficient in undermining the public health goal, and that consumption of sugary drinks fall after imposition of taxes, though demand is inelastic. We argue that inelasticity of demand for SSB may require a combination of interventions to sufficiently reduce excess consumption of sugar drinks.

## Background

 Sugar sweetened beverage (SSB) consumption has been highlighted as one of the main drivers of the growing burden of non-communicable diseases (NCDs). Scholars have highlighted several alternative ways of reducing SSBs consumption and taxation has emerged as one of the most prominent ones, with the World Health Organization (WHO) describing taxation of SSBs as the “best buy for health” in addressing NCDs. Several countries, both high-income and low- and middle-income have introduced SSB taxes. An obvious concern is the extent to which such policies will be effective in reducing SSB consumption and whether this will be sustained for a long time. Clearly, the extent of effectiveness of such policies will not only depend on how consumer preferences are altered by the taxes so that they reduce consumption, but also how producers respond to such taxes. Forde et al^1^ have recently published an article based on qualitative Interviews where they attempt to uncover how producers respond to sugar taxes. They argue that these responses could be important “spillover effects” that may undermine public health goals and classify these spillover effects in terms of “four Ps” or “marketing mix,” namely price, product, promotion, and placement. These four Ps. Imply that not only can the producer change the price, but they could also change the product through reformulation, they could also change promotional or advertising, and lastly how the product is placed. This article discusses some of the key issues that arise in their paper and sheds more light on the complex question of how to design SSB policies to achieve the desired results.

## Main Discussion

 In evaluating the paper by Forde and colleagues,^1^ our main argument is that they raise important questions, which we believe could be a good starting point for more rigorous research. Our position is based on five main arguments, namely that: (1) highlighted company responses may not be sufficient to undermine the public health goal; (2) tax on products that did not reformulate still leads to lower SSB consumption, but elasticity is low; (3) a combination of interventions is needed to reduce SSB consumption; (4) there is no clear quantitative evidence that SSB taxes elicit an Increase In advertising; and (5) a mixed method design is needed to understand producer responses and to what extent may dilute the public health goals. We discuss each of these in return.

###  Highlighted company responses may not be sufficient to undermine the public health goal.

 Forde et al^1^ are coherent in their discussion of how producers may reformulate their products in response to SSB taxes. However, despite a clear motivation in the introduction, they do not discuss what aspects of reformulation or other market responses may be thought to undermine public health goals and which ones may not. We think that two of the six company responses (changing messaging and change in distribution, placement and packaging) may undermine the public health goal while the other four (reformulation, increased product price, reduce portion size, and developing new products) are more likely to reinforce it. When the product messaging is changed to show consumers that they have reformulated to care about the health of the public when in fact this reformulation is not a substantial reduction in sugar content, the public may be misled by such message. This may result in increased consumption rather than reduction, which on the overall, may result in increased caloric intake. Change in distribution, placement and packaging may also mislead the public. Forde et al^1^ highlight that some of their interviewees said “large multinational companies might have recoupled lost UK sales following taxation by increasing sales elsewhere.” Additionally, the findings that some brands responded by repackaging lower sugar variants to resemble high sugar variants, appears to be aimed at misleading the public and this may undermine the public health goal.

 Nonetheless, we think that these two aspects that may undermine the public health goal are not likely to be as strong as the aspects that reinforce it, namely reformulation, increased product price, reduce portion size, and developing new products. All these factors result in lower caloric intake. Evidence in the United Kingdom^2^ and South Africa^3^ based on quantitative data before and after the tax show that reformulation where companies reduced the amount of sugar content in the beverages to reduce tax liability substantially decreased population caloric intake. As evidence of reformulation in the United Kingdom, for example, lower SSB tax were collected.

###  Tax on products that do not reformulate still leads to lower SSBs consumption, but elasticity is low.

 Sugar taxes have been demonstrated to substantially reduce consumption and ultimately caloric intake in countries such as Mexico, the United Kingdom, South Africa, and the United States. Additionally, even with ex-ante and ex-post product reformulation, purchases of sugar drinks have been demonstrated to fall, not just in modelling studies,^4^ but also in empirical work and these effects persist in the medium to long term.^5^ For products that were affected by the tax in the United Kingdom, for example — those that did not reformulate sufficiently — the tax liability was fully shifted to consumers resulting in a substantial reduction in SSB consumption by around 18%.^2^ Although this reduction is substantial, it points to inelastic demand for these sugar drinks that did not reformulate (as in demand reducing by less than the proportionate increase in price). We think the theoretical framework by Forde and colleagues^1^ is helpful in understanding why the elasticity of demand for products that where not reformulated may be low. Their framework describes context-specific factors where “high brand” companies are less likely to reformulate because they enjoy brand loyalty. High brand companies are likely to have loyal customers and this may explain the low elasticity in Dickson et al^2^ context. Nonetheless, the aspect of brand loyalty as determining elasticity is not new in the economics and marketing literature. Thus, we believe their study could have been more informative if it was formulated with a mixed method design so that they could collect data from the firms they were interviewing — and a few more to increase sample size and external validity — to check which market responses were significant and prevalent.

 The full important of the relatively inelastic demand raises the question of why governments do not raise sugar taxes sufficiently high to achieve the required reductions in demand. However, this is not straight forward as sugar tax policy has serious political economy dimensions where there are arguments that pursuing public health goals through sugar taxes may harm livelihoods through job losses.^6^

###  A combination of interventions is needed to reduce SSB consumption.

 Given high brand strength of some products and that customers may be loyal to certain SSBs, those who can afford will still pay even when prices increase. This means that to achieve higher reductions in excess sugar consumption, there is need for a combination of interventions. Public health campaigns could also be key. In an evaluation of mass media campaigns to reduce consumption of SSBs in three cities in the United States, Farley et al^7^ found a statistically significant 4.1% decrease in soda sales in intervention areas. It would be nice to see the extent to which SSB consumption or sales would fall if such public health campaigns where combined with sugar taxes or if public health campaigns were implemented in different ways.

###  Quantitatively, there is no clear evidence that SSB taxes lead to change in advertising.

 Based on anecdotal evidence, Forde et al^1^ argue that in response to the introduction of SSB taxes, producers more frequently increase strategies such as changing messages, distribution, and/or placement. There are limited or no studies that have quantitatively shown this. However, without having collected data on advertising activities or expenditure from their interviewees, it is unclear whether these producers significantly increase marketing activities as they indicate. On the contrary, there is strong evidence elsewhere showing that SSB taxes, in fact, led to a fall in promotional activities. For example, a quasi-experimental study found that price promotions in supermarkets for SSBs that were taxed reduced substantially when Oakland introduced a SSB tax,^8^ and they explained that this could have been due to manufacturers trying to save costs as a results of the tax. Product reformulation and marketing changes may impose extra costs and coupled with uncertainty of whether such reformulations or marketing changes would be effective, producers may be reluctant to substantially increase marketing activities. Similarly, another study in Seattle found a reduction in supermarket interior market displays following the introduction of a SSB tax.^9^

**Figure 1:**
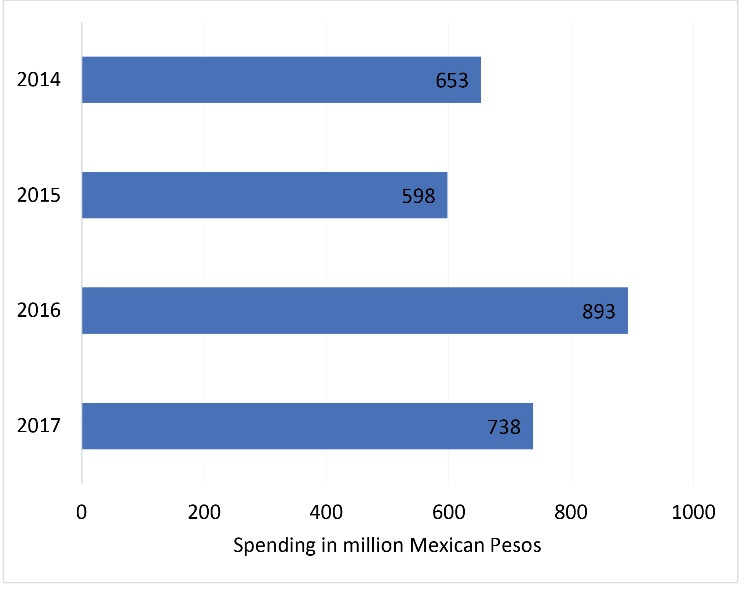
Trend in Advertising Spend for the Coca-Cola Company – Mexico.Source: Author own computation based on data from the 2021 Coca-Cola annual report.

 Perhaps an important aspect to look at is whether, and the extent to which, SSB producers may have increased promotions and advertising in response to SSB taxation is to look at year-on-year changes in advertising expenditure on SSB. We do not have access to UK specific advertising expenditure for SSBs even for one company. But we can focus on Mexico which introduced an SSB tax in 2014. Data from the Coca-Cola Company in Mexico (Figure 1) shows that their advertising expenditure in Mexico fell the year following the SSB tax in 2015 before increasing in 2016 and falling again in 2017 (Statistica, 2017).

 Further, we have extracted global advertising figures for Coca-Cola for the period 2015-2021. Given the wave and heated debates on taxing sugary drinks, we would expect to see significant increases in advertising expenditure by Coca-Cola if advertising is used to counter advances in policies on sugar taxes. The figure does not suggest so. While this trend may have been due to other factors that influence advertising expenditure other than the introduction of SSB taxes, it re-enforces the idea that the effect of SSB taxes on advertising expenditure is unclear and yet to be fully explored. Simply analyzing the trend in advertising expenditures could be misleading as the changes could have been attributed to a myriad of other factors. There is thus need for the researcher to provide more robust studies to collaborate the narrative that there is significant increased marketing and advertising post SSB taxation in the United Kingdom that could counteract the effect of SSB taxes.

**Figure 2:**
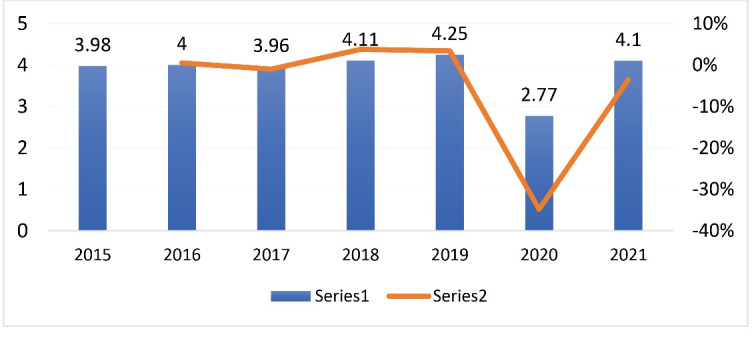
Coca-Cola Company Advertising Expenditure in Billions (USD). Source: Author own computation based on data from the 2021 Coca-Cola annual report.

## Conclusion

 With the proliferation of SSB taxes particularly in countries with high SSB consumption, producers of SSBs have had varied responses with unclear impacts on the effectiveness of these taxes. The authors provide a framework for evaluating the responses of producers of SSBs to these taxes, however, there are still a number of questions that remain unanswered and this forms the basis for this commentary. The authors identified reformulation of SSBs as one of the main responses to taxation. It must be acknowledged however that this may actually reinforce public health gains if reformulation reduces the sugar content of their products. Further, while the framework proposed by the authors provides an explanation for the price inelasticity of demand of SSBs that are not reformulated, the authors could have adopted a mixed methods approach and incorporated quantitative data from the firms they interviewed to check which market responses were significant and prevalent. While the authors indicate the need for a combination of SSB taxes and public health interventions, the extent to which SSB consumption or sales would fall in scenarios where additional public health interventions are implemented was not discussed. This could include information on the types of interventions and their complementary effect on the reduction of SSB consumption and the choice of an optimum combination. The authors further indicate that there is limited quantitative evidence to support the hypothesis that SSB taxes lead to changes in advertising, whereas we find that literature and statistics indicate a fall in advertising expenditure following the implementation of an SSB tax.

## Ethical issues

 Not applicable.

## Competing interests

 Authors declare that they have no competing interests.
